# Resistance and virulence features of hypermucoviscous *Klebsiella pneumoniae* from bloodstream infections: Results of a nationwide Italian surveillance study

**DOI:** 10.3389/fmicb.2022.983294

**Published:** 2022-08-15

**Authors:** Fabio Arena, Giulia Menchinelli, Vincenzo Di Pilato, Riccardo Torelli, Alberto Antonelli, Lucia Henrici De Angelis, Marco Coppi, Maurizio Sanguinetti, Gian Maria Rossolini

**Affiliations:** ^1^Department of Clinical and Experimental Medicine, University of Foggia, Foggia, Italy; ^2^Microbiology and Virology Unit, University Hospital “Riuniti,”, Foggia, Italy; ^3^Istituti di Ricovero e Cura a Carattere Scientifico (IRCCS) Don Carlo Gnocchi ONLUS, Florence, Italy; ^4^Dipartimento di Scienze di Laboratorio e Infettivologiche, Fondazione Policlinico Universitario A. Gemelli Istituti di Ricovero e Cura a Carattere Scientifico (IRCCS), Rome, Italy; ^5^Department of Surgical Sciences and Integrated Diagnostics (DISC), University of Genoa, Genoa, Italy; ^6^Department of Experimental and Clinical Medicine, University of Florence, Florence, Italy; ^7^Microbiology and Virology Unit, Florence Careggi University Hospital, Florence, Italy; ^8^Department of Medical Biotechnologies, University of Siena, Siena, Italy; ^9^Dipartimento di Scienze Biotecnologiche di Base, Cliniche Intensivologiche e Perioperatorie, Università Cattolica del Sacro Cuore, Rome, Italy

**Keywords:** genome sequencing, clones, carbapenemases, virulence factor, multi-drug resistant

## Abstract

Among Enterobacterales, *Klebsiella pneumoniae* (Kp) is one of the major opportunistic pathogens causing hospital-acquired infections. The most problematic phenomenon linked to Kp is related to the dissemination of multi-drug resistant (MDR) clones producing carbapenem-hydrolyzing enzymes, representing a clinical and public health threat at a global scale. Over the past decades, high-risk MDR clones (e.g., ST512, ST307, ST101 producing *bla*_KPC–type_ carbepenemases) have become endemic in several countries, including Italy. Concurrently, the spread of highly virulent Kp lineages (e.g., ST23, ST86) able to cause severe, community-acquired, pyogenic infections with metastatic dissemination in immunocompetent subjects has started to be documented. These clones, designated as hypervirulent Kp (hvKp), produce an extensive array of virulence factors and are highly virulent in previously validated animal models. While the prevalence and distribution of MDR Kp has been previously assessed at local and national level knowledge about dissemination of hvKp remains scarce. In this work, we studied the phenotypic and genotypic features of hypermucoviscous (HMV, as possible marker of increased virulence) Kp isolates from bloodstream infections (BSI), obtained in 2016–17 from 43 Italian Laboratories. Antimicrobial susceptibility testing, whole genome sequencing and the use of two animal models (*G. mellonella* and murine) were employed to characterize collected isolates. Over 1502 BSI recorded in the study period, a total of 19 Kp were selected for further investigation based on their HMV phenotype. Results showed that hvKp isolates (ST5, ST8, ST11, ST25) are circulating in Italy, although with a low prevalence and in absence of a clonal expansion; convergence of virulence (yersiniabactin and/or salmochelin, aerobactin, regulators of mucoid phenotype) and antimicrobial-resistance (extended-spectrum beta-lactamases) features was observed in some cases. Conventional MDR Kp clones (ST307, ST512) may exhibit an HMV phenotype, but with a low virulence potential in the animal models. To the best of our knowledge, this work represents the first systematic survey on HMV and hvKp in Italy, employing a functional characterization of collected isolates. Future surveillance programs are warranted to monitor the threatening convergence of virulence and resistance among MDR Kp and the spread of hvKp.

## Introduction

At the beginning of the 80’s, bacteria of genus *Klebsiella* had mostly been studied as a cause of community-acquired pneumonia, occurring particularly in chronic alcoholics (Carpenter, 1990). More recently, *Klebsiella* spp., and especially *K. pneumoniae* (Kp) have been recognized as a major public health threat due to their ability to accumulate antimicrobial-resistance determinants and cause severe infections in the healthcare setting. Kp is currently considered the prototype of opportunistic, hospital-acquired, multi-drug resistant (MDR) pathogen (Munoz-Price et al., 2013; Tacconelli et al., 2018).

Some Kp lineages, called hypervirulent *K. pneumoniae* (hvKp), are associated with a more virulent behavior (Shon et al., 2013). hvKp is an evolving pathotype that is more virulent than classical Kp due to the combination of several features including: K1, K2 or K5 capsule polysaccharide; the presence of horizontally acquired virulence factors encoding the siderophores aerobactin (Iuc) and salmochelin (Iro), and the genotoxin colibactin (Clb); a hypermucoid phenotype (often associated with the presence of regulators of the mucoid phenotype (namely Rmp) (Russo and Marr, 2019; Zhu et al., 2021). Although incompletely predictive of a virulent behavior, the presence of the hypermucoid phenotype (HMV) is commonly used as a phenotypic laboratory marker for identification of hvKp strains (Shon et al., 2013; Catalán-Nájera et al., 2017).

hvKp usually cause community-acquired infections in individuals who are previously healthy (Choby et al., 2020). Infections are more common in the Asian Pacific Rim but are occurring globally (Shon et al., 2013; Russo and Marr, 2019). hvKp infections frequently involve multiple sites or subsequently exhibit metastatic spread, requiring source control. hvKp have an increased ability to cause central nervous system infection and endophthalmitis, which require rapid recognition and site-specific treatment (Choby et al., 2020). hvKp are rarely MDR and most strains retain susceptibility to most of available antibiotics (Shon et al., 2013; Russo and Marr, 2019; Zhu et al., 2021). In fact, MDR Kp strains responsible of hospital acquired infections and hvKp strains causing community acquired infections apparently belong to two distinct and well segregated bacterial populations (Bialek-Davenet et al., 2014; Wyres et al., 2020). However, convergence of virulence and resistance determinants within hybrid lineages is possible and has been increasingly reported worldwide (Tang et al., 2020). Various carbapenemase genes have recently been detected in hvKp isolates, including those encoding enzymes of the OXA-48, KPC, NDM, and VIM lineages, generating great concern at international and European level (European Centre for Disease Prevention and Control (ECDC), 2021). At least one fatal hospital outbreak of MDR hvKp strains has been reported in China, where carbapenemase-producing hvKp are increasingly common (Gu et al., 2018).

In Italy, sporadic cases of infections caused by HMV Kp have been documented (Arena et al., 2016a; Piazza et al., 2022), in some cases the isolates showed a MDR phenotype associated with carbapenemase production (Arena et al., 2017; Scaltriti et al., 2020), but detailed knowledge about the epidemiology of hvKp strains is lacking.

In this work, we undertook a nationwide surveillance to investigate the epidemiology and features of HMV Kp isolated from bloodstream infections in Italy, a setting of endemic dissemination of MRD Kp clones (Di Pilato et al., 2021). The collected isolates were subjected to comprehensive molecular characterization and were also studied for pathogenicity using the *Galleria mellonella* and two different mouse infection models.

## Materials and methods

### Study design and isolates collection

**FIGURE 1:**
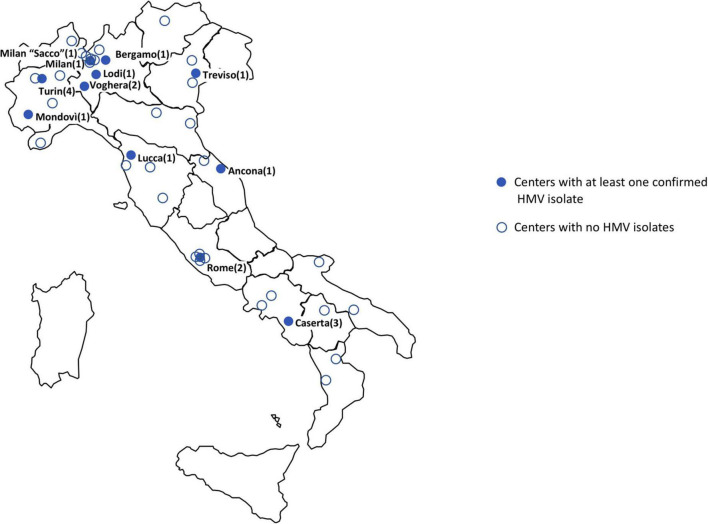
Geographic distribution of participating laboratories. Blue dots represent centers with at least one HMV isolate. Empty dots represent centers with absence of HMV or non-confirmed HMV isolates only (number of confirmed HMV isolates per center in brackets).

For a 6 months period (1st December 2016–31st May 2017), 43 laboratories located in different areas of Italy (Figure 1) and belonging to the KHIN (*Klebsiella pneumoniae* Hypermucoviscous Italian Network of AMCLI) were asked to collect and store all consecutive, non-duplicated, Kp isolates from blood cultures showing a positive “string test” (HMV) (Fang et al., 2004). A dedicated slide set for the correct “string test” performance and interpretation was elaborated and distributed to each center together with the participation letter. Bacterial identification was performed, at participating Laboratories, using routine methods (biochemical methods and/or MALDI-ToF). Laboratories were also asked to collect and report the following data: (i) total number of beds in the hospital/s served by the Laboratory; (ii) total number of blood culture sets collected during the study period; (iii) number of Kp bacteremia episodes occurred during the study period (Supplementary Table 1). Suspected HMV Kp were stored at –80°C until shipment. At the end of the collection period all isolates categorized as HMV by satellite centers were centralized to the Microbiology Laboratory of the Department of Experimental and Clinical Medicine, University of Florence (Florence, Italy) for HMV phenotype confirmation and further analyses (central Laboratory).

### Antibiotic susceptibility testing

Susceptibility of confirmed HMV isolates to amikacin, cefepime, ceftazidime, ceftazidime/avibactam, ceftolozane/tazobactam, ciprofloxacin, colistin, gentamicin, meropenem, piperacillin/tazobactam, and tigecycline was carried out by broth microdilution (ISO 20776–1, 2019) using lyophilized custom plates (Merlin Diagnostika, Germany). Fosfomycin susceptibility was tested by agar-dilution in the presence of 25 mg glucose-6-phosphate (Camarlinghi et al., 2019). Results were interpreted according to the European Committee on Antimicrobial Susceptibility Testing (EUCAST breakpoints v 12.0). For tigecycline the PK-PD breakpoint of 1 mg/L was adopted.

### Whole genome sequencing and bioinformatics

Genomic DNA of confirmed HMV isolates was extracted using the DNeasy PowerLyzer PowerSoil Kit (Qiagen, Hilden, Germany) and subjected to whole genome sequencing (WGS) with the MiSeq platform (Illumina Inc., San Diego, CA), using a 2 × 250 paired-end approach (Arena et al., 2020). Draft genome assemblies were generated using SPAdes v3.11 (Bankevich et al., 2012).

The PathogenWatch online platform (by Center for Genomic Pathogen Surveillance)^1^ —was used to generate genotyping report of all sequenced genomes. The software automatically passes assembled genomes to Kleborate and Inctyper pipelines. The Kleborate software provides in depth species, ST (according to the 7 genes MLST scheme), virulence genes, capsule typing, O locus typing, and antimicrobial resistance prediction. Kleborate examines five key acquired virulence loci that are associated with invasive infections and are found at high prevalence among hvKp strains: the siderophores yersiniabactin (ybt), aerobactin (iuc) and salmochelin (iro), the genotoxin colibactin (clb), and the hypermucoidy locus *rmpADC*. The alternative hypermucoidy marker gene *rmpA2* is also searched. Kleborate outputs a simple categorical virulence score, and if resistance screening is enabled, an antimicrobial resistance score as well. These scores provide a rough categorization of the strains to facilitate monitoring resistance-virulence convergence. The virulence score ranges from 0 to 5: 0 = negative for all of yersiniabactin (ybt), colibactin (clb), aerobactin (iuc) 1 = yersiniabactin only 2 = yersiniabactin and colibactin (or colibactin only) 3 = aerobactin (without yersiniabactin or colibactin) 4 = aerobactin with yersiniabactin (without colibactin) 5 = yersiniabactin, colibactin and aerobactin. Kleborate screens input genomes against a curated version of the CARD database of acquired resistance gene alleles, and groups these by drug class for reporting purposes. The resistance score ranges from 0 to 3: 0 = no ESBL, no carbapenemase (regardless of colistin resistance) 1 = ESBL, no carbapenemase (regardless of colistin resistance) 2 = Carbapenemase without colistin resistance (regardless of ESBL genes or OmpK mutations) 3 = Carbapenemase with colistin resistance (regardless of ESBL genes or OmpK mutations) (Wyres et al., 2016; Wick et al., 2018; Lam et al., 2021). Inctyper is an in-house tool that uses the PlasmidFinder database and BLAST to identify the contigs containing a plasmidic Inc reference gene (Carattoli et al., 2014). The presence of core- and accessory virulence factors was investigated using the Virulence Factor Database (VFDB; last access on May 20, 2022) (Chen et al., 2005). For isolates encoding multiple virulence genes, manual analyses were conducted using NCBI BLAST tool to screen for the presence of additional determinants associated with canonical pK2044-/pLVpK-like virulence plasmids [e.g., genes coding for proteins involved in iron metabolism (cobW) and transport (fecI-fecA), the hemin and lysine transport system (shiF), metabolic transporter (peg-344), and transcriptional regulations of virulence gene expression (luxR)]. Adjunctive single target analyses were performed using the NCBI BLAST tool with the database BIGSdb curated by Pasteur Institute.^2^

### Lethality in the *Galleria mellonella* model

Bacterial strains were grown in LB broth, aerobically, at 35 ± 2°C, harvested during exponential phase (OD600≈0.7), and washed once with 10 mM phosphate-buffered saline (PBS, pH 6.5). Bacteria were then suspended in PBS to an OD600 of 1.5, corresponding approximately to 1 × 10^9^ CFU/mL. Larvae weighing 450–600 mg were used for the experiments. For comparative evaluation of virulence, groups of 10 larvae were injected with 5 × 10^5^ CFU of each strain and with sterile PBS as control. Larvae were kept at 35 ± 2°C in the dark, in humidified atmosphere, with food, and daily examined for pigmentation and mobility. Time of death was recorded at 24, 48, and 72 h. For each strain, data from three independent experiments were combined. GraphPad Prism 6.0 software was used for mean mortality ± SD calculation (GraphPad Software Inc., La Jolla, CA). *K. pneumoniae* NTUH-H2044, an ST23 hvKp strain and the less virulent ST258 Kp KKBO-1 strain were also included in all experiments as references (Fang et al., 2004; Arena et al., 2016b).

### Murine model

Isolates were grown on TSA agar. A single colony was suspended in 10 ml of Brain Heart Infusion broth and incubated over-night at 35 ± 2°C. Overnight cultures were centrifuged, and pellets were resuspended in PBS at final concentrations of 10^9^ CFU/mL. A total of 100 μl of each strain were injected into 10 BALB/c mice (Harlan Italy S.r.l., Udine, Italy) through the tail vein. All animals used for experiments were females 10 weeks of age weighing approximately 20–25 g. Forty-eight hours after inoculation, mice were sacrificed by cervical dislocation. Kidneys and livers were removed, weighed, and homogenized using a stomacher (model 80; Pbi International, Milan, Italy). Serial homogenate dilutions were plated onto Brain Heart Infusion agar for CFU count. To confirm their identity and to avoid possible contaminations, different morphologies’ isolates were identified using the MALDI-ToF based method. Animal experiments were performed at animal facilities of the Catholic University (Rome, Italy), under a protocol approved by the Institutional Animal Use and Care Committee at Università Cattolica del S. Cuore, Rome, Italy and authorized by the Italian Ministry of Health, according to Legislative Decree 116/92, which implemented the European Directive 86/609/EEC on laboratory animal protection in Italy. Animal welfare was routinely checked by veterinarians of the Service for Animal Welfare, (Protocol n°935/2017-PR).

### Statistics

Differences between groups of isolates with different virulence levels in the animal models were assessed using the one-way ANOVA. Analysis was performed using Stata 15 (StataCorp, College Station, TX) or GraphPad Prism 7 (GraphPad Software, San Diego, CA) software, and *P* < 0.05 was considered statistically significant.

## Results

During the study period, the 43 participating Laboratories (serving a total of 29,719 hospital beds) processed 191,800 blood culture sets. A total of 1,502 bacteremia episodes sustained by bacteria identified as Kp were reported. Of these, 52 (52/1,502, 3.5%) were reported as “string test positive” according to satellite Laboratory screening. At the central Laboratory, 19 isolates were confirmed as HMV (19/1,502, 1.3% of HMV Kp bacteremia cases) and subjected to further analysis. Most of confirmed HMV isolates were obtained in Laboratories from northern Italy, mainly from Lombardy (6/19 isolates, 31.6%) (Figure 1). Prevalence of HMV strains over the total of Kp BSI episodes at participating centers ranged from 0 to 20% (Supplementary Table 1).

### Antimicrobial susceptibility of confirmed hypermucoviscous isolates

Overall, the most active drugs were ceftazidime/avibactam (5.3% of resistant isolates), followed by amikacin, meropenem, gentamicin, colistin and fosfomycin (10.6, 21.0, 36.8, 36.8, and 47.4% of resistant isolates, respectively). Five (5/19, 26.3%) isolates showed a meropenem MIC > 0.125 mg/L and were therefore considered as suspect carbapenemase producers. Eleven isolates (11/19, 57.9%) had an MIC for ceftazidime > 1 mg/L and were suspect ESBL producers (Table 1, Supplementary Table 2).

**TABLE 1:** Antimicrobial susceptibility testing results for HMV isolates included in the study.

Antibiotics	MIC range	MIC50	MIC90	% S	% I	% R
Amikacin	=4–32	=4	8	89.4	0.0	10.6
Cefepime	=1– > 8	4	>8	42.1	5.3	52.6
Ceftazidime	=0.25– > 32	32	>32	42.1	0.0	57.9
Ceftazidime/ avibactam	=1/4– > 8/4	=1/4	2/4	94.7	0.0	5.3
Ceftolozane/ tazobactam	=1/4– > 8/4	2/4	>8/4	52.6	0.0	47.4
Ciprofloxacin	=0.0625– > 8	2	>8	42.1	0.0	57.9
Colistin	=1– > 8	=1	>8	63.2	0.0	36.8
Fosfomycin	=4– > 128	16	>128	52.6	0.0	47.4
Gentamicin	=0.25–32	=0.25	>32	63.2	0.0	36.8
Meropenem	=0.125– > 16	=0.125	>16	73.7	5.3	21.0
Piperacillin- tazobactam	2/4– > 128/4	16/4	>128/4	42.1	0.0	57.9
Tigecycline	1– > 8	2	8	36.8	0.0	63.2

MICs are expressed in μg/mL.

### Genome sequencing and virulence data

#### Bacterial identification and clonality

Based on the nature of the housekeeping beta-lactamase gene, 18 isolates were identified as Kp (presence of a *bla*_SHV_-type gene) and one as *Klebsiella variicola* (presence of a *bla*_LEN_-type gene). According to the 7 genes MLST scheme, one third of Kp isolates (6/18) belonged to ST307, three isolates were assigned to ST512 and two isolates to ST29. The remaining were singletons of ST5, ST8, ST11, ST25, ST35, and ST198. One isolate represented a new ST: a single locus variant of ST37 (new *phoE* allele).

#### ST307 isolates

**FIGURE 2:**
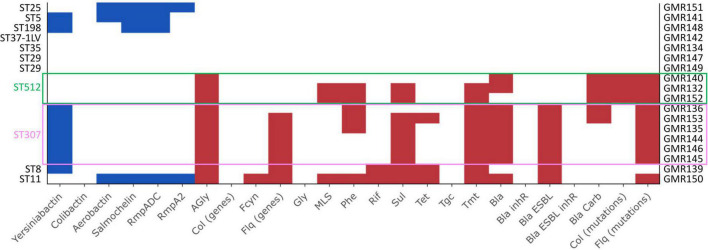
Results of Kleborate pipeline visualized with the Kleborate-viz tool. Detected resistance determinants are in red while virulence factors are in blue. Legend: AGly_acquired (aminoglycosides), Bla_acquired (beta-lactamases), Bla_inhR (beta-lactamases with resistance to beta-lactamase inhibitors), Bla_Carb (carbapenemase), Bla_ESBL (extended spectrum beta-lactamases), Bla_ESBL_inhR (extended spectrum beta-lactamases with resistance to beta-lactamase inhibitors), Fcyn (fosfomycin), Flq (fluoroquinolones), Gly (glycopeptides), MLS (macrolides), Phe (phenicols), Rif (rifampin), Sul (sulfonamides), Tet (tetracyclines), Tmt (trimethoprim), Tgc (tigecycline).

All the six ST307 isolates had a KL102 capsule locus type (associated with *wzi173* allele) with a O1/O2v2 O locus and carried the yersiniabactin siderophore gene cluster *ybt 9*, associated with a ICE*Kp3* (Figure 2, Supplementary Figure 1, and Supplementary Table 3). With respect to the resistome, two isolates carried a *bla*_KPC_ carbapenemase gene, a *bla*_KPC–3_, and a *bla*_KPC–2_ variant, respectively. One isolate (GMR153) encoded a CMY-16, class C, beta-lactamase. All isolates encoded a CTX-M-15 ESBL and trimethoprim-sulfamethoxazole resistance determinants. All except one carried genes coding for multiple aminoglycoside-modifying enzymes and fluoroquinolones resistance (Figure 2 and Supplementary Table 3).

All isolates were phenotypically resistant to ceftazidime and ciprofloxacin. Five isolates were colistin resistant and two were resistant to carbapenems (the two KPC-type producers) (Supplementary Table 2).

**FIGURE 3:**
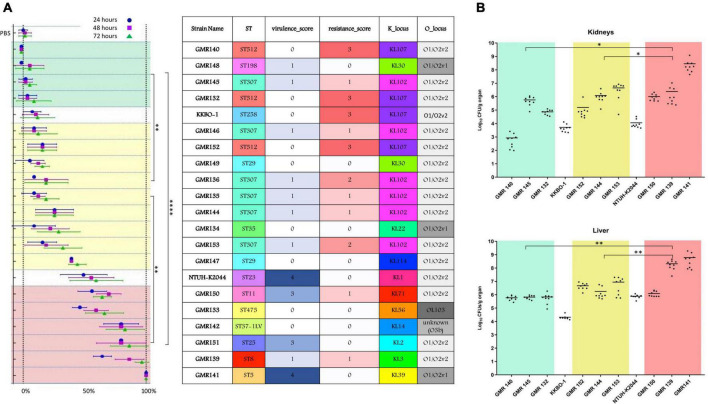
Summary of most relevant features of studied strains (ST, virulence score, resistance score, K type and O type) and results obtained in the animal infection models. **(A)** Results of experiments in the *G. mellonella* model expressed as mortality rate (percentage of dead over the total of inoculated larvae). Means and SD of three replicate experiments are show at different time points for each strain (after 24, 48, and 72 h). **(B)** Results of virulence experiments, for representative HMV isolates, in the murine model expressed as Log10CFU/g organ of the studied strains, recovered from kidneys and livers. Each dot represents data obtained from one mouse specimen and the solid line represents the mean value. In both the models, the NTUH-K2044 virulent strain and the KKBO-1 low-virulence strain were included and used as reference for classification of studied isolates in three different virulence categories: low (green zone), intermediate (yellow) and high (red). Mortality rate at 72 h **(A)** and Log10 counts **(B)** for the three groups were compared. *P*-values < 0.05 were considered significant; *< 0.05, **<0.005, ****<0.0001.

In the *G. mellonella* animal model, all ST307 isolates showed a low/intermediate virulence potential, comparable to that of the control strain KKBO-1 (Figure 3A). These results were confirmed in both murine infection models (Figure 3B).

#### ST512 isolates

The three isolates belonging to ST512 had a KL107 capsule locus type (associated with *wzi154* allele) and a O1/O2v2 O locus. None of the searched virulence factors was found. All isolates showed a complex content of resistance genes (resistance score: 3), including a *bla*_KPC–3_ carbapenemase, aminoglycosides resistance determinants, mutational alterations previously associated with colistin resistance (alterations in the *mgrB* gene) and fluoroquinolones resistance. Two out of three isolates encoded also for trimethoprim-sulfamethoxazole resistance determinants (Figure 2, Supplementary Figure 1, and Supplementary Table 3).

Coherently, all isolates were resistant to ceftazidime, cefepime, ceftolozane-tazobactam, piperacillin-tazobactam and meropenem. One isolate was resistant to ceftazidime/avibactam also (GMR140). Two isolates out of three were colistin resistant and all were resistant to fosfomycin and ciprofloxacin (Supplementary Table 2).

The virulence potential of these strains was low in two cases and intermediate in one case (GMR152) in both *G. mellonella* and murine infection models (Figures 3A,B).

#### ST29 isolates

The two ST29 isolates were associated with a O1/O2v2 O locus but carried a different K loci, KL30 (*wzi*85) and KL114 (*wzi354*), respectively. None of the virulence and resistance determinants included in the analysis pipeline was detected (Figure 2, Supplementary Figure 1, and Supplementary Table 3). Both isolates were phenotypically susceptible to all tested drugs, except for GMR149 that was resistant to fosfomycin and showed a tigecycline MIC above the PK/PD breakpoint (MIC > 8 μg/mL) (Supplementary Table 2).

In the animal models, ST29 isolates showed a level of virulence that was intermediate between that of KKBO-1 and NTUH-K2044 strains (Figures 3A,B).

#### Other sequence types

The ST5 isolate (GMR141) was associated with the KL39 (*wzi112* allele) and the O1/O2v1 O locus. The isolate carried an *ybt 2* locus associated with a ICE*Kp1* (Ybt ST324), together with synthesis loci for salmochelin (*iro3 lineage—SmST 3*), aerobactin (*iuc1*), and regulator of hypermucoidy (*rmpADC* lineage 3). Sequence analysis revealed that the acquisition of the former virulence factors was not likely associated with the presence of a pK2044-like element, the archetypal virulence plasmid from the hypervirulent *K. pneumoniae* NTUH-K2044, suggesting that a novel or recombined virulence element could have mediated the acquisition of these traits. Concerning the resistome, the housekeeping SHV-11 beta-lactamase only (a non-ESBL) was found (Figure 2, Supplementary Figure 1, and Supplementary Table 3), coherently with the susceptibility profile (Supplementary Table 2). In the animal models, GMR141 was the most virulent strain of the collection, with lethality and ability to infect mouse liver and kidneys higher than the virulent control NTUH-K2044 (Figures 3A,B).

The ST8 isolate (GMR139) included in the study was associated with a KL3 (*wzi59* allele) and O1/O2v2 capsular locus and O locus, respectively. The genome was characterized by the presence of *ybt 7* locus associated with a ICE*Kp7*. By contrast, the content of resistance determinants was relevant with a *bla*_SHV–2_ (ESBL phenotype), multiple aminoglycosides modifying enzymes, trimethoprim-sulfamethoxazole resistance genes and an acquired *qnr*-type gene associated with fluoroquinolones resistance (Figure 2, Supplementary Figure 1, and Supplementary Table 3). The isolate was resistant to ceftazidime, cefepime, ceftolozane-tazobactam, piperacillin-tazobactam and ciprofloxacin and was highly virulent in the *G. mellonella* and the murine models (Figures 3A,B).

The ST11 isolate (GMR150) was associated with a KL71 capsular locus (*wzi72*) and a O3b locus. The aerobactin (*iuc 1*), salmochelin (*iro 1*) and the regulators of hypermucoidy (*rpmADC* locus) were detected. Sequence analysis revealed that a plasmid element resembling pK2044 (query coverage: 84%, BLAST identity: 99.6%) likely contributed to the acquisition of the virulence factors (i.e., aerobactin, salmochelin, *rmp* cluster). Concerning the resistome, genes coding for an acquired class C (*bla*_DHA–1_) and class A (*bla*_CTX–M–65_) beta-lactamases, as well as for multiple acquired aminoglycoside modifying enzymes and for fosfomycin resistance (*fosA3*) were detected (Figure 2, Supplementary Figure 1, and Supplementary Table 3). The isolate was phenotypically resistant to ceftazidime, cefepime, ceftolozane-tazobactam, ciprofloxacin, fosfomycin and piperacillin-tazobactam and was highly virulent in the *G. mellonella* and murine models, with lethality higher than NTUH-K2044 (Figures 3A,B).

The ST25 isolate (GMR151) was associated with a KL2 capsular locus (*wzi72*) and a O1/O2v2 O locus. The content of virulence genes was relevant (virulence score: 3) including synthesis loci for salmochelin (*iro1 lineage*), aerobactin (*iuc1*), and regulators of the mucoid phenotype (*rmpADC*). As for the ST11 isolate, sequence analysis revealed the presence of a pK2044 derivative plasmid (query coverage: 81%, BLAST identity: 99.5%) that likely contributed to the acquisition of former virulence factors (i.e., *iuc1*, *iro1*, *rmpADC*).

Except the housekeeping SHV-11 beta-lactamase, none of the screened resistance determinants was found (Figure 2 and Supplementary Table 3), coherently with the susceptibility profile (Supplementary Table 2). A high lethality in the *G. mellonella* model was shown (Figure 3A).

The ST198 isolate (GMR148) was associated with a KL30 capsular locus (*wzi85*) and a O1/O2v1O locus, carried an *ybt 2* locus associated with a ICE*Kp1* (Ybt ST324); it carried a variant of the salmochelin synthesis locus (*iro 3* lineage) and regulator of hypermucoidy (*rmpADC* lineage 3). The acquisition of the former virulence factors was not likely associated with the presence of a pK2044-like element. No acquired resistance genes were detected, and the isolate was phenotypically susceptible to tested antibiotics (Supplementary Table 2). The virulence level in the *G. mellonella* model was extremely low (Figure 3A).

The ST35 (GMR134), the new ST single locus variant of ST37 (GMR142), and the *K. variicola* (GMR133) isolates were associated with KL22 (wzi37)/O1/O1v1, KL14 (*wzi14*)/unknown (closest match to O3b) and KL56/OL103, capsular/O locus types, respectively. No resistance genes nor virulence determinants were found and the isolates were susceptible to all tested drugs, except for two isolates (GMR134, GMR142) that were resistant to fosfomycin (Figure 2 and Supplementary Tables 2, 3). The three isolates showed a variable level of virulence: intermediate (GMR134) or high (GMR142 and GMR 133).

## Discussion

In this work we report on the results of the first Italian nationwide surveillance (more than 40 hospitals involved) study for HMV Kp bacteremia. Overall, the prevalence of isolates with an HMV phenotype (positive string test), over the total of Kp obtained from blood cultures, at the involved centers, was low (19/1502, 1.3%). A trend trough an overestimation of the HMV phenotype was observed in most of the participating centers. In fact, satellite centers stored 52 suspected HMV isolates but 19 only were confirmed HMV at the central Laboratory. This observation underscore that need of a certain level of Laboratory skills for the correct interpretation of the “string test.”

Of the 19 HMV confirmed isolates, only six were highly virulent in the animal models and could be therefore considered hvKp (overall prevalence of highly virulent HMV lineages: 0.4%), confirming that HMV phenotype and hypervirulence can’t be considered synonymous in Kp (Catalán-Nájera et al., 2017). Systematic surveillance studies are lacking but similar low prevalence data were obtained from other countries (Peirano et al., 2013; Örsten et al., 2020) with the notable exception of China, Taiwan and South Korea. Indeed, HMV Kp circulation is historically more relevant in these areas (Lee et al., 2006, 2017; Li et al., 2014; Zhang et al., 2016).

In our surveillance study, despite the low prevalence, at least one HMV isolate was found in 11/43 centers located in different geographic areas of the Country, mainly clustered in Northern Italy, underscoring a significant geographic dissemination of the phenomenon.

The antimicrobial susceptibility profile of collected HMV strains reflected the situation reported for Italy in the same period. In particular, the 26.3% of HMV strains included in our surveillance was meropenem non-susceptible (suspect carbapenemase-producers) and the 57.9% were ceftazidime non-susceptible (suspect ESBL producers). In 2017, the EARS-NET European Surveillance System (European Center for Disease Prevention and Control), reported for Italy similar prevalence data with: 32.1 and 56.1% of carbapenems non-susceptible and 3rd generation cephalosporins non-susceptible Kp isolates, respectively.^3^

Our study also provides the first comprehensive overview of genetic, phenotypic and virulence features of systematically collected HMV strains, from Italy.

Analyzing the data, it was possible to identify three different patterns of isolates. Pattern i) including isolates that accumulated multiple resistance determinants (including carbapenemase genes and colistin resistance mutations) but with absence or a low content of virulence genes and a low/intermediate virulence behavior in animal models. These isolates belong to two major high-risk clones, namely ST512 and ST307.

A multi-national large genomic surveillance study revealed that the population of carbapenem-resistant Kp, from invasive infections, in Italy was mainly contributed by ST258/512, ST101, and ST307 nosocomial clones producing the KPC-type carbapenemase, during 2013–14 (David et al., 2019).

Consistently, a more recent Italian survey performed in the 2016–2017 period (Di Pilato et al., 2021) showed that ST512 was largely prevalent among carbapenemase producers, followed by ST307, both producing the KPC-type beta-lactamases., Therefore, we can speculate that occasional acquisition events of the HMV phenotype may be recognized within the endemically disseminated nosocomial clones circulating in Italy. A case describing an HMV Kp belonging to the ST515 epidemic clone has been reported from Northern Italy. In that case, despite the low virulence potential in the *G. mellonella*, the isolate caused a fatal systemic infection with liver abscesses in an immunocompromised patient (Arena et al., 2017).

Interestingly, similarly to what previously described, none of the isolates in this pattern was HMV due to the presence of the classic regulators of the mucoid phenotype (*rmpADC*). Consistently, none of these isolates carried classic or recombined versions of most common virulence plasmids.

As such, the presence of *rmp* genes as molecular marker predictive of the HMV phenotype should be carefully considered for epidemiological purposes. The pathogenetic significance of the HMV phenotype in these cases remain uncertain. However, none of pattern (i) isolates was highly virulent in animal models. Differently from what previously described by other authors (Diago-Navarro et al., 2014), in the HMV population included in this study, the mortality in *G. mellonella* model predicted high virulence in mice models. Our study supports the use of the *G. mellonella* model as a feasible and predictive approach for high throughput screening of virulence behavior in Kp.

Pattern (ii) including isolates coupling the absence of major resistance and virulence determinants with a low or intermediate virulence potential in the animal model. These strains belonging to ST29 and ST35 were phenotypically multi-susceptible and the HMV phenotype wasn’t linked to *rmpADC* mediated mechanism.

Pattern (iii) including isolates characterized by a relevant content of virulence genes and, in some cases, presence of multiple resistance determinants and an MDR phenotype with retained susceptibility to carbapenems. These strains, belonging to ST5, ST8, ST11, and ST25, were all highly virulent in animal models.

According to previously published extensive genomic data, ST5 and ST8 isolates are extremely infrequent (David et al., 2019).

GMR141, an ST5 strain, had the highest virulence potential of the collection and showed multi-susceptible phenotype. It carried the most extensive array of virulence factors (virulence score 4) including: ICEKp1, the aerobactin, the salmochelin siderophores and a *rmpADC* hypermucoidy lineage 3 (Figure S1). In the genomic population study conducted in 2019 by David et al. (2019), three ST5 strains only, over a total of 1,717 Kp, were described and none of these strains displayed a such relevant content of virulence genes. Therefore, we can conclude that this strain represents a potentially emerging new virulent clone.

The same can be asserted for GMR139, an ST8 ESBL strain with the *ybt7* cluster and a virulent behavior in the animal models. ST8 isolates are quite uncommon (one isolate over 1,717 only in the David et al. study). Interestingly, GMR139 has a KL3 *cps* type that has been previously identified as a relevant virulence factor for *Klebsiella pneumoniae* subsp. *rhinoscleromatis* (Corelli et al., 2018).

Differently from the previous two strains included in this cluster, GMR150 belongs to a well-known MDR high-risk clone, namely ST11. ST11 has been previously associated with virtually all carbapenemase classes, mainly *bla*_KPC–2_, *bla*_NDM–1_, and *bla*_OXA–48_ (Liao et al., 2020). Some ST11 lineages have been previously associated with an aggressive behavior and hospital outbreaks of carbapenem-resistant ST11 have been described. A KPC-2-producing hvKp ST11 has been linked to a cluster of severe lung infection with fatal multi-organ failure or septic shock in two Chinese hospitals (Gu et al., 2018; Xu et al., 2019). Similar to previously characterized ST11 hvKp MDR strains, GMR150 carries a plasmid highly correlated to pK2044, found in NTUH-K2044, that likely contributed to the acquisition of the former virulence factors (i.e., aerobactin, salmochelin, *rmp* cluster). As other ST11 MDR strains from China, GMR150 was highly virulent in the *G. mellonella* and murine models (Zhang et al., 2020). Differently from previously described ST11 hvKp MDR strains, our isolate carried an unusual capsular type: the KL71. KL71 has been previously linked to *Klebsiella variicola* (de Campos et al., 2021). Even if the strain described in this work was carbapenem-susceptible and none of most common carbapenemase classes was detected, the content of acquired resistance mechanisms was notable, including a *bla*_CTX–M–65_ a *bla*_DHA–1_ beta-lactamases and the *fosA3* fosfomycin resistance gene. Interestingly, these two latter resistance mechanisms are strongly epidemiologically linked to the Asiatic continent (Taniguchi et al., 2017; Hennequin et al., 2018; Hao et al., 2021; Wang et al., 2022). To our best knowledge, this is the first description of a FosA3 producing Kp reported from Italy. Therefore, we can conclude that we documented the presence of a highly virulent ST11 MDR Kp clone with several genetic features all supporting a link with the Asiatic continent. Analyzing the features of this strain, the classic proposed paradigm depicting a situation with a clear segregation between populations of MDR and hvKp isolates seems to be surpassed (Bialek-Davenet et al., 2014).

The last strain included in this pattern belongs to the ST25 (GMR151). ST25, associated with the KL2 capsule, is a known hypervirulent strain initially linked to outbreaks of septicemia cases in pigs in England (Bidewell et al., 2018; Wanford et al., 2021). Recently, an expansion of hypermucoviscous MDR ST25 (production of *bla*_KPC_ carbapenemase) has been reported from Argentina (Cejas et al., 2019). As for the ST11 isolate, the presence of a pK2044 derivative plasmid was likely the explanation of the virulence potential of this strain.

Finally, some isolates were not assimilable to the proposed patterns. For example, the ST198 was avirulent in the animal models but carries an array of virulence genes resembling that of the ST5 strain with the notable exception of the aerobactin siderophore. The new ST single locus variant of ST37 (GMR142) was multi-susceptible too but highly virulent and presented the new O locus (O3b-like).

The only *K. variicola* (GMR133) isolate in our collection was highly virulent in the *G*. *mellonella* model and presented a multi-susceptible phenotype. *K. variicola* has been associated with severe infections (Rodríguez-Medina et al., 2019) and hypervirulent strains have been described (Lu et al., 2018). Interestingly, our strain didn’t carry classic regulators of the mucoid phenotype nor plasmids similar to those previously associated with the HMV phenotype in *K. variicola* (Rodríguez-Medina et al., 2020).

Concluding, in this work we describe the genetic features of HMV Kp isolates circulating in Italy using an approach based on a first phenotypic screening with the “string test” followed by genome sequencing and final confirmation of the virulent behavior in the *G. mellonella* and mouse models. The approach enabled us to demonstrate the presence in the Italian territory of MDR (mainly associated with an ESBL phenotype), highly virulent clones (such as ST11) characterized by the carriage of derivates of known virulence plasmid. Furthermore, we identified some new highly virulent clones belonging to ST8 and ST5. Adjunctively, we confirmed the presence of carbapenem-resistant ST512 and ST307 HMV isolates with a high content of resistance genes and a low/intermediate virulence level. A possible limitation of our study is that, due to the study design, patients demographic information, and comorbidities data were not collected and analyzed.

Overall, our data can be considered reassuring. In fact, the prevalence of hvKP was extremely low and all highly virulent and MDR strains in our collection were singletons of different sequence types (STs). Apparently, a clonal expansion of most problematic lineages was not present. Furthermore, the classic hvKp clones (ST23 and ST86) were not found.

However, the prevalence of hvKp was higher in some centers, distributed in northern Italy, that could become hot spots of dissemination. Continued efforts in identifying and tracking the dissemination of hvKp are warranted to deliver timely public health responses aimed at containing their spread within the healthcare system.

## Data availability statement

The datasets presented in this study can be found in online repositories. The names of the repository/repositories and accession number(s) can be found below: https://www.ncbi.nlm.nih.gov/, PRJNA781811.

## Ethics statement

The animal study was reviewed and approved by the Institutional Animal Use and Care Committee at Università Cattolica del S. Cuore (Protocol n°935/2017-PR).

## The AMCLI *Klebsiella pneumoniae* hypermucoviscous Italian network

Maria Grazia Cusi (Siena University Hospital, Siena, Italy), Anesi Adriano (Maggiore Hospital, Lodi, Italy), Maria Labonia (IRCCS “Casa Sollievo della Sofferenza,” San Giovanni Rotondo, Foggia, Italy), Ivana Vada (Cardinal Massaia Hospital, Asti, Italy), Francesca Maldera (Della Murgia F. Perinei Hospital, Altamura, Bari, Italy), Sandra Carpiceci (S. Pietro Fatebenefratelli Hospital, Rome, Italy), Chiara Vettori, Romano Mattei (USL North Western Tuscany, Lucca, Italy), Tamara Brunelli (USL Center Tuscany, Prato, Italy), Antonietta Sinno (Madonna delle Grazie Hospital, Matera, Italy), Sara Rimoldi (Sacco Hospital, Milan, Italy), Barbara Pieretti (Santa Croce Hospital, AO Riuniti Hospital Marche Nord, Ancona, Italy), Richard Aschbacher (Bolzano Central Hospital, Azienda Sanitaria dell’Alto Adige, Bolzano, Italy), Vincenzo Minasi, Loredana Delflorio (Multimedica S.p.a., Sesto San Giovanni, Milan, Italy), Andrea Bartolini, Margherita Scapaticci (San Camillo Hospital, Treviso, Italy), Maria Zoppelletto (ASL 3 Bassano de Grappa, Vicenza, Italy), Cinzia Rossi, Claudia Canale (Castelli Hospital -Asl VCO, Verbania, Italy), Pier Andrea Dusi (USL 1 Imperiese, San Remo, Imperia, Italy), Maria Maddalena Grossi (S. Francesco Hospital, Paola, Cosenza, Italy), Maria Federica Pedna (AUSL della Romagna, Pievesistina, Cesena, Italy), Bruno Mariani (San Camillo Forlanini Hospital, Rome, Italy), Chiara Vincenzi (Riuniti Hospital Ancona, Ancona, Italy), Gerardino Amato, Linda Degl’Innocenti (Cardarelli Hospital, Naples, Italy), Erminio Torresani, Elisabetta Cesana (Istituto Auxologico Italiano, Milan, Italy), Domenico Salamone (USL North Western Tuscany, Pontedera, Pisa, Italy), Maria Paola Ferrero (Koelliker Hospital, Turin, Italy), Daniela Rossi, (Villa Salus Hospital, Mestre, Venice, Italy), Fulvia Milano (ASL Vercelli, Vercelli, Italy), Luigi Principe (A. Manzoni Hospital, Lecco, Italy), Roberto D’Angelo, Loredana Vizzini (A.S.P. Golgi-Redaelli, Milan, Italy), Marco Passera (ASST Papa Giovanni XXIII, Bergamo, Italy), Claudia Venturelli (AOU Policlinico di Modena, Modena, Italy), Floriana Gona (San Raffaele Scientific Istitute, Milan, Italy), Erminia Casari (Humanitas Research Hospital, Milan, Italy), Rita Greco, Vittorio Panetta (AORN Sant’Anna e San Sebastiano, Caserta, Italy), Lara Ines Bellazzi (ASST di Pavia—Voghera Civile Hospital, Pavia, Italy), Maria Carmela Cava, Carmen Luciana Bonanno (Sandro Pertini Hospital-ASL Roma 2, Rome, Italy), Alfredo Focà, Angela Quirino (AOU Mater Domini, Catanzaro, Italy), Maria Rosaria Catania (AOU Federico II di Napoli, Naples, Italy), Davide Carcione (Centro cardiologico Monzino IRCCS, Monza Brianza, Italy), Lucina Fossati, Gabriele Bianco, Rossana Cavallo (A.O. Città della Salute e della Scienza, Presidio Molinette, Turin, Italy), Carla Fontana (Tor Vergata University Hospital, Rome, Italy), Michela Quatela (Mondovì Hospital, Cuneo, Italy).

## Author contributions

FA: conceptualization, formal analysis, investigation, data curation, writing—original draft, writing—review and editing, and project administration. GM and VD: investigation, data curation, and writing—review and editing. RT, LH, and MC: investigation. AA: investigation and writing—review and editing. MS: resources, writing—review and editing, and supervision. GR: resources, draft, writing—review and editing, and supervision. All authors contributed to the article and approved the submitted version.

## Footnotes

1
https://pathogen.watch

2
https://bigsdb.pasteur.fr/klebsiella/

3
https://www.ecdc.europa.eu/en/surveillance-atlas-infectious-diseases

## Supplementary material

Supplementary Figure 1 Distribution of core and acquired factors involved in host-pathogen interaction and virulence (source: The virulence factor database, VFDB; last access on May 20, 2022). Details of gene products (right panel) were also reported in alphabetical order.

Supplementary Table 1 List of included centers with information on number of hospital beds, blood cultures sets, Kp bacteremia episodes, suspected and confirmed HMV bacteremia per year.

Supplementary Table 2 Antimicrobial susceptibility testing results of confirmed HMV isolates.

Supplementary Table 3 Analytic Kleborate output of confirmed HMV isolates subjected to WGS.
