# Supplementary figures and images for: Resistance and virulence features of hypermucoviscous Klebsiella pneumoniae from bloodstream infections: Results of a nationwide Italian surveillance study
Source: Front Microbiol. 2022 Aug 15;13:983294. doi: 10.3389/fmicb.2022.983294 (PMC9531727; doi:10.3389/fmicb.2022.983294)

 Presence

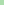 Absence

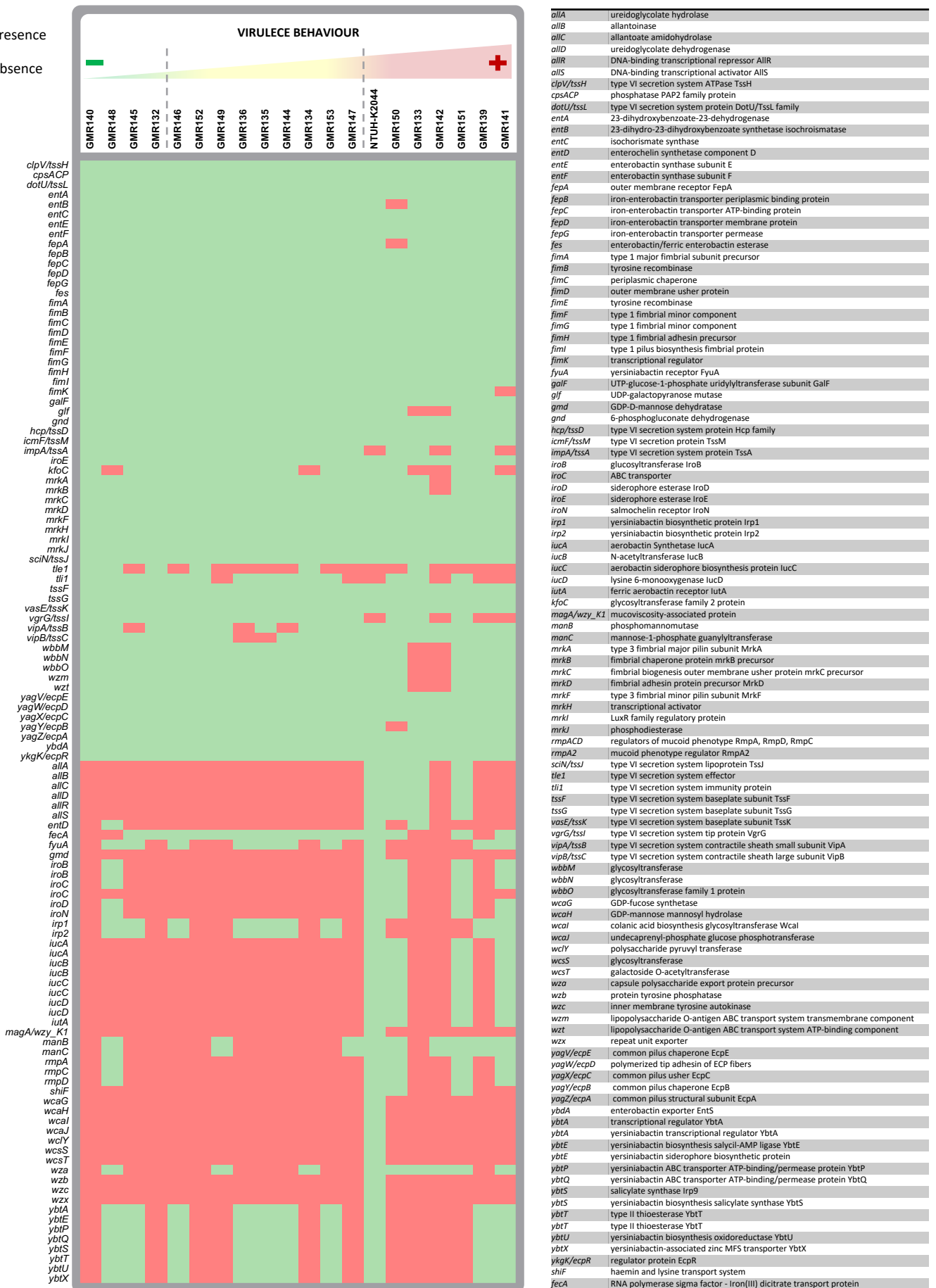

Supplement: Supplementary Figure 1 — Distribution of core and acquired factors involved in host-pathogen interaction and virulence (source: The virulence factor database, VFDB; last access on May 20, 2022). Details of gene products (right panel) were also reported in alphabetical order. [file Data_Sheet_1.PDF]
