# Supplementary material for: Resistance and virulence features of hypermucoviscous Klebsiella pneumoniae from bloodstream infections: Results of a nationwide Italian surveillance study
Source: Front Microbiol. 2022 Aug 15;13:983294. doi: 10.3389/fmicb.2022.983294 (PMC9531727; doi:10.3389/fmicb.2022.983294)
Supplement: Supplementary Table 1 — List of included centers with information on number of hospital beds, blood cultures sets, Kp bacteremia episodes, suspected and confirmed HMV bacteremia per year. [file Table_1.DOCX]

| **Supplementary Table 1** | | | | | | | |  |
| --- | --- | --- | --- | --- | --- | --- | --- | --- |
| **Center name** | **City** | **Center ID.** | **N. beds** | **N. blood-culture sets** | **N. of *K. pneumoniae* bacteraemia episodes** | **N. of blood-cultures positive *K. pneumoniae* suspected HMV*** | **N. of blood-cultures positive *K. pneumoniae* confirmed HMV^#^** | **Prevalence (%)** |
| Siena University Hospital | Siena | 1 | 737 | 3524 | 16 | 0 | 0 | 0 |
| Maggiore Hospital | Lodi | 3 | 600 | 9374 | 6 | 2 | **1** | **16.7** |
| IRCCS "Casa Sollievo della Sofferenza" | S. Giovanni Rotondo (Foggia) | 4 | 850 | 5013 | 124 | 0 | 0 | 0 |
| Cardinal Massaia Hospital | Asti | 5 | 497 | 1615 | 15 | 0 | 0 | 0 |
| Della Murgia F. Perinei Hospital | Altamura (Bari) | 7 | 230 | 267 | 8 | 0 | 0 | 0 |
| S. Pietro Fatebenefratelli Hospital | Rome | 8 | 436 | 1437 | 9 | 0 | 0 | 0 |
| USL North Western Tuscany | Versilia (Lucca) | 10 | 415 | 4801 | 41 | 0 | 0 | 0 |
| USL Center Tuscany | Prato | 12 | 600 | 9777 | 17 | 0 | 0 | 0 |
| Madonna delle Grazie Hospital | Matera | 14 | 370 | 1700 | 7 | 0 | 0 | 0 |
| Sacco Hospital | Milan | 15 | 1500 | 5400 | 5 | 1 | **1** | **20** |
| USL North Western Tuscany | Lucca | 17 | 1026 | 8565 | 50 | 2 | **1** | **2** |
| Santa Croce Hospital | Marche Nord (Ancona) | 18 | 592 | 2115 | 8 | 0 | 0 | 0 |
| Bolzano Central Hospital, Azienda Sanitaria dell'Alto Adige | Bolzano | 19 | 660 | 9600 | 30 | 0 | 0 | 0 |
| Multimedica S.p.a. | Sesto San Giovanni (Milan) | 20 | 779 | 1275 | 20 | 0 | 0 | 0 |
| San Camillo Hospital | Treviso | 21 | 114 | 186 | 6 | 2 | **1** | **16.7** |
| ASL 3 Bassano de Grappa | Bassano del Grappa (Vicenza) | 22 | 503 | 3726 | 29 | 0 | 0 | 0 |
| Castelli Hospital -Asl VCO | Verbania-Pallanza | 23 | 310 | 644 | 3 | 0 | 0 | 0 |
| USL 1 Imperiese | San Remo (Imperia) | 25 | 574 | 4673 | 48 | 0 | 0 | 0 |
| S. Francesco Hospital | Paola (Cosenza) | 26 | 125 | 162 | 28 | 1 | 0 | 0 |
| AUSL della Romagna | Pievesistina (Cesena) | 27 | 3201 | 12481 | 128 | 0 | 0 | 0 |
| San Camillo Forlanini Hospital | Rome | 29 | 830 | 4867 | 54 | 22 | **2** | **3.7** |
| Riuniti Hospital Ancona | Ancona | 30 | 934 | 6182 | 99 | 1 | **1** | **1** |
| Cardarelli Hospital | Naples | 31 | 900 | 4031 | 66 | 0 | 0 | 0 |
| Istituto Auxologico Italiano | Cusano Milanino (Milan) | 32 | 150 | 574 | 7 | 3 | 0 | 0 |
| USL North Western Tuscany | Pontedera (Pisa) | 33 | 240 | 970 | 23 | 0 | 0 | 0 |
| Koelliker Hospital | Turin | 35 | 150 | 13 | 0 | 0 | 0 | 0 |
| Villa Salus Hospital | Mestre (Venice) | 37 | 200 | 326 | 0 | 0 | 0 | 0 |
| ASL Vercelli | Vercelli | 38 | 380 | 1856 | 15 | 0 | 0 | 0 |
| A. Manzoni Hospital | Lecco | 39 | 593 | 3547 | 13 | 3 | 0 | 0 |
| A.S.P. Golgi-Redaelli | Milan | 40 | 1400 | 264 | 1 | 0 | 0 | 0 |
| ASST Papa Giovanni XXIII | Bergamo | 41 | 1036 | 847 | 28 | 1 | **1** | **3.6** |
| AOU Policlinico di Modena | Modena | 42 | 1111 | 17031 | 30 | 0 | 0 | 0 |
| San Raffaele Scientific Istitute | Milan | 43 | 1355 | 9693 | 181 | 1 | **1** | **0.5** |
| Humanitas Research Hospital | Milan | 44 | 750 | 3170 | 31 | 0 | 0 | 0 |
| AORN Sant'Anna e San Sebastiano | Caserta | 46 | 550 | 5000 | 15 | 3 | **3** | **20** |
| ASST di Pavia - Voghera Civile Hospital | Voghera (Pavia) | 49 | 500 | 1114 | 22 | 3 | **2** | **13.6** |
| Sandro Pertini Hospital-ASL Roma 2 | Rome | 50 | 420 | 14002 | 169 | 0 | 0 | 0 |
| AOU MATER DOMINI | Catanzaro | 52 | 230 | 566 | 4 | 1 | 0 | 0 |
| AOU Federico II di Napoli | Naples | 55 | 800 | 6462 | 4 | 0 | 0 | 0 |
| Centro cardiologico Monzino IRCCS | Milan | 56 | 180 | 1023 | 0 | 0 | 0 | 0 |
| A.O. Città della Salute e della Scienza, Presidio Molinette | Turin | 57 | 1700 | 5300 | 84 | 5 | **4** | **4.8** |
| Tor Vergata University Hospital | Rome | 58 | 420 | 14002 | 38 | 0 | 0 | 0 |
| Mondovì Hospital | Mondovì (Cuneo) | 59 | 771 | 4625 | 20 | 1 | **1** | **5** |
| Total n. |  |  | 29719 | 191800 | 1502 | 52 | **19** | **1.3** |

*: sent as HMV by satellite center; # :confirmed as HMV at central Laboratory.
